# Production and Characterization of Recombinant Single-Chain Variable Fragment (scFv) Antibody Against *Fasciola gigantica* Saposin-like Protein 2

**DOI:** 10.3390/ijms27104474

**Published:** 2026-05-16

**Authors:** Komsil Rattanasroi, Apichai Prachasuphap, Panadda Dhepakson, Supanan Chansap, Pornanan Kueakhai, Narin Changklungmoa

**Affiliations:** 1 Faculty of Allied Health Sciences, Burapha University, Long-Hard Bangsaen Road, Mueang District, Chonburi 20131, Thailand; 61810040@go.buu.ac.th (K.R.); supanan.cn@go.buu.ac.th (S.C.); pornanan@go.buu.ac.th (P.K.); 2 Department of Medical Sciences, Medical Life Sciences Institute, Tiwanond Road, Nonthaburi 11000, Thailand; apichai.p@dmsc.mail.go.th (A.P.); panadda.d@dmsc.mail.go.th (P.D.); 3 Research Unit for Vaccine and Diagnosis of Parasitic Diseases, Burapha University, Long-Hard Bangsaen Road, Mueang District, Chonburi 20131, Thailand

**Keywords:** recombinant antibody, single-chain variable fragment (scFv), saposin-like protein 2, *Fasciola gigantica*

## Abstract

Saposin-like protein 2 (SAP2) exhibits strong immunogenicity as an antigen for immunodiagnosis in ruminant and human fasciolosis. Most available immunodiagnostic test kits are based on polyclonal and monoclonal antibodies against antigens from *Fasciola* spp. Previous studies demonstrated that polyclonal and monoclonal antibodies against SAP2 showed high specificity and could effectively detect *Fasciola* spp. infections at an early stage. However, polyclonal antibodies are extremely difficult to produce, and quality control is not possible during production; the procedure also involves considerable financial investment. To overcome these problems, we developed a single-chain variable fragment (scFv) to control quality in each production cycle and reduce the cost of manufacturing immunodiagnostic kits. Our objectives were to produce and characterize an scFv that binds the SAP2 from the liver fluke *Fasciola gigantica*. We constructed the scFv by genetic engineering: we cloned immunoglobulin genes and linked them with flexible polypeptide linkers composed of repeating glycine and serine residues. We selected an scFv with high affinity for binding SAP2 using the phage-display technique and produced it using a prokaryotic expression system. The scFv was characterized via in silico and in vitro methods to confirm its specificity for SAP2, including IMGT/V-QUEST, IMGT/Collier-de-Perles, HADDOCK 2.4, ELISA, immunoblotting, and immunohistochemistry. The scFv was successfully produced and purified using Ni-NTA affinity chromatography. The purified scFvFgSAP2 was approximately 27 kDa, as confirmed by SDS-PAGE and immunoblot analysis. An indirect ELISA and immunoblotting indicated that scFvFgSAP2 had strong reactivity with *F. gigantica* compared to other parasite species. Moreover, immunolocalization of scFvFgSAP2 confirmed that it binds specifically to natural SAP2 in the cecal epithelium cells of *F. gigantica*. Therefore, this scFv targeting SAP2 is an effective material and can be used to develop immunodiagnostic procedures.

## 1. Introduction

Fasciolosis, a disease caused by infection with the liver flukes *F. gigantica* and, to a lesser extent, *F. hepatica*, occurs worldwide. It leads to anemia, chronic inflammation, bile duct obstruction, cholangitis, and liver abscesses in infected individuals and livestock [1,2,3]. Fasciolosis is associated with high morbidity and mortality rates, resulting in significant economic losses in the livestock industry [4,5,6]. The survival of *Fasciola* spp. depends on their ability to extract nutrients from the host, for which they employ a complex combination of excretory–secretory (ES) products that facilitate tissue invasion, immune evasion, and nutrient digestion [7]. Among the many proteins found in these ES products are the highly active proteases cathepsin L, cathepsin B, fatty acid binding protein, thioredoxin (Trx), glutathione S-transferase (GST), superoxide dismutase (SOD), and saposin-like protein 2 (SAP2) [8,9]. In addition to digesting the proteins in blood and tissues that the parasite needs for survival, these proteases also break down host tissues, facilitating easier migration and penetration [10]. Additionally, to maintain parasite persistence, ES components actively modulate the immune response.

Saposin-like proteins (SAPs) are a diverse family of lipid-interacting proteins, including more than two hundred proteins from a broad range of organisms, from the most distant primitive protozoa to mammals [11]. In previous studies on *F. hepatica*, recombinant FhSAP2 proteins were shown to induce lysis of human erythrocytes and peripheral blood mononuclear cells (PBMCs) [12]. In *F. gigantica*, three SAPs encoding cDNA (FgSAP-1, FgSAP-2, and FgSAP-3) have been reported [13,14,15]. SAP2 is an essential protein for the survival of certain pathogens. By contributing to host tissue invasion and protein degradation, it plays a crucial role in pathogenesis [13]. Parasites meet their nutritional needs by secreting protease enzymes, which proteolytically break down hemoglobin. SAP2 has become a highly interesting molecular target for the development of new diagnostic and therapeutic tools, as it plays a crucial role in the parasite’s life cycle and is expressed early in infection [16,17,18,19].

Current test kits for the diagnosis of *Fasciola* spp. infection are based on antigens from the tegument or ES products of *Fasciola* spp., including SAP2 [17,18,19], cathepsin L1 (CatL1) [20,21], cathepsin L1H (CatL1H) [22,23,24], cathepsin B3 (CatB3) [25], fatty acid-binding protein (FABP) [26,27,28], and tegument-associated protein 28.5 kDa antigen [29]. Most available immunodiagnostic test kits utilize a polyclonal or monoclonal antibody against these antigens to detect early-stage *Fasciola* spp. infections [30,31,32]. Although established hybridoma cell lines can provide consistent antibody yields, the initial development process necessitates animal immunization and can be highly resource-intensive, which contributes to the overall manufacturing costs of diagnostic kits. Therefore, in this study, we focus on using genetic engineering to produce single-chain variable fragment (scFv) antibodies that specifically bind to SAP2. This approach offers advantages over traditional hybridoma techniques, including reduced animal use, lower production costs, improved quality control, and scalability for industrial production [33,34].

These scFv antibodies can be used to develop cost-effective diagnostic kits and in other biotechnological applications. The genetic engineering process involves randomly combining variable regions of antibody heavy and light chains with linker peptides to create diverse antibody libraries [35]. These libraries are then displayed on phage pIII proteins for bio-panning, a selection process that identifies scFv antibodies with high specificity and affinity for the target antigen. This versatile technique is widely used in both basic and advanced research, with numerous applications in the biotechnology industry [35,36,37,38]. The development of this recombinant monoclonal antibody against SAP2 will yield biotechnological applications for combating fasciolosis.

## 2. Results

### 2.1. Expression and Purification of rFgSAP2

**Figure 1:**
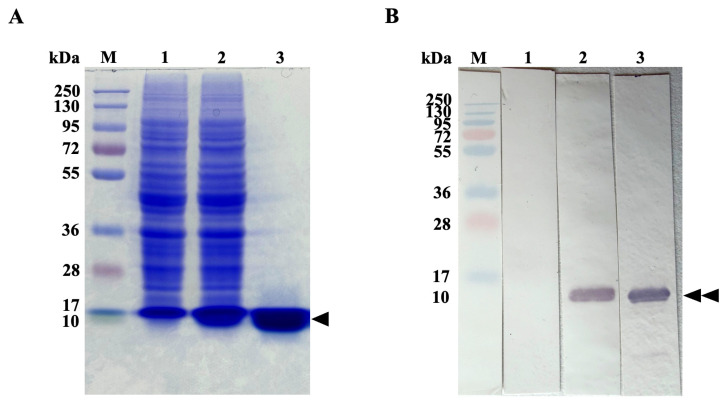
(**A**) SDS-PAGE analysis of expression and purification of rFgSAP2. PageRuler Plus Prestained Protein Ladder (lane M). Proteins from non-induced condition (lane 1); whole lysate after induction (lane 2); recombinant FgSAP2 remains after purification (lane 3). The triangle indicates the rFgSAP2 protein at approximately 10 kDa. (**B**) Immunoblot analysis using an anti-rFgSAP2 confirms the identity of the purified approximately 10 kDa rFgSAP2 (Double black triangles).

The rFgSAP2 protein was successfully expressed in *Escherichia coli* (*E. coli*) BL21(DE3) and purified by Ni-NTA affinity chromatography. The rFgSAP2 purity and molecular weight were evaluated by SDS-PAGE, revealing a distinct band at approximately 10 kDa (Figure 1A). The identity of the rFgSAP2 was further validated by immunoblot analysis, which detected a specific immunoreactive band at the expected molecular weight (Figure 1B).

### 2.2. Determination of Immune Response

**Figure 2:**
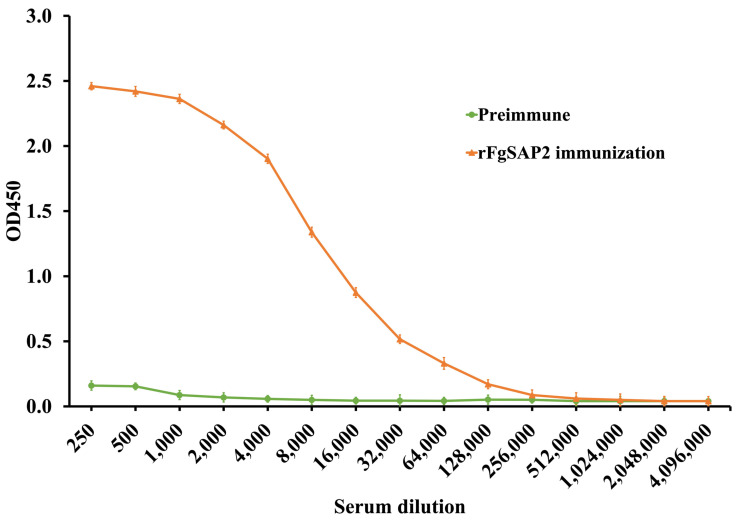
Serum antibody titers determined by indirect ELISA after immunization. The Titer was observed ten days after the second booster.

The level of immune response in serum was determined by indirect ELISA ten days after the second booster immunization. The antibody titer was approximately 256,000, as shown in Figure 2, indicating a strong immune response.

### 2.3. Cloning and Construction of scFvFgSAP2 Library

**Figure 3:**
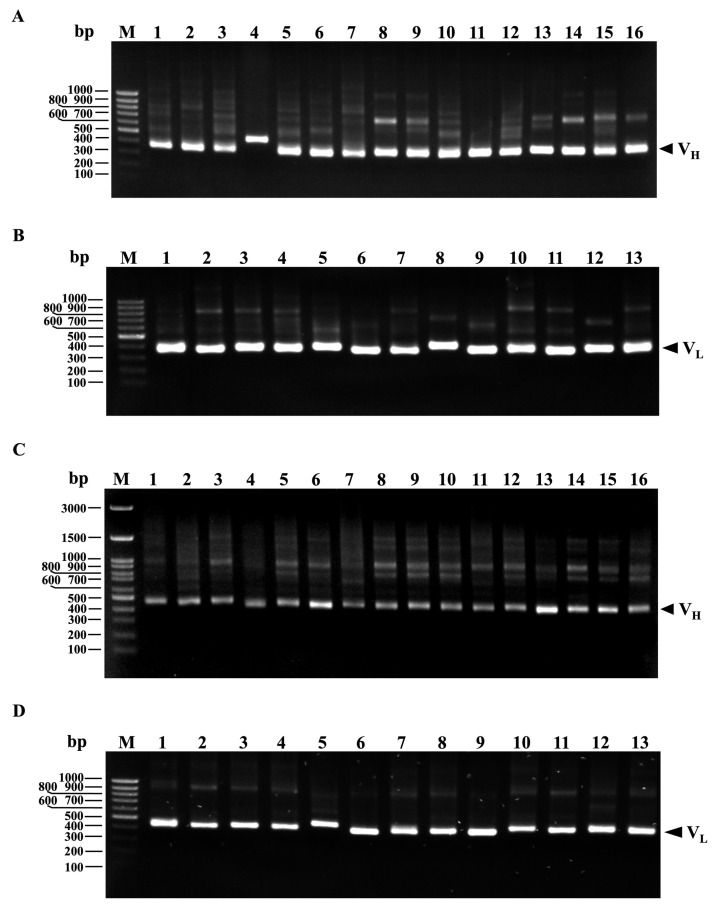
Agarose gel electrophoresis analysis. PCR amplification of VH gene repertoires (**A**) including GeneRuler DNA Ladder (Lane M) and amplified VH gene using different forward primers (Lane 1–16). PCR amplification of VL gene repertoires (**B**) including amplified VL gene using different forward primers (Lane 1–13). Reamplification of VH gene repertoires with (Gly4Ser)3 linker and restriction sites (**C**), including VH gene repertoires with (Gly4Ser)3 linker and restriction sites using different forward and reverse primers (Lane 1–16). Reamplification of VL gene repertoires with (Gly4Ser)3 linker and restriction sites (**D**), including VL gene repertoires with (Gly4Ser)3 linker and restriction sites using different forward and reverse primers (Lane 1–13).

**Figure 4:**
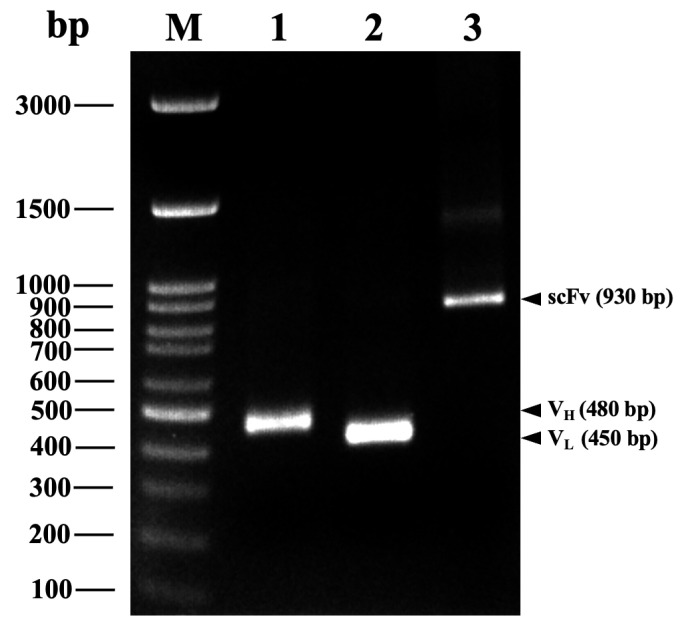
Agarose gel electrophoresis analysis. GeneRuler DNA Ladder (lane M). PCR amplification of variable heavy chain (VH; lane 1) and light chain (VL; lane 2) domains. scFvFgSAP2 was linked by glycine–serine linker (lane 3).

scFvFgSAP2 was constructed by genetic engineering: immunoglobulin variable heavy chains (VH) and light chains (VL) against SAP2 were cloned from first-strand cDNA. The VH gene repertoires were amplified by PCR using a set of VH forward primers paired with a mixture of JH reverse primers. Similarly, the VL gene repertoires were amplified using a set of VL forward primers paired with a mixture of JK reverse primers. The PCR products were analyzed by 1.5% agarose gel electrophoresis. Bands corresponding to the 340–480 bp VH and 320–450 bp VL fragments were excised (Figure 3A,B). The purified VH gene fragments were reamplified using a set of forward primers containing an *Sfi*I site and reverse primers incorporating a (G4S)2 linker. Similarly, the VL gene fragments were reamplified using forward primers with a (G4S)2 linker and reverse primers containing a *Not*I site. The PCR products were analyzed by 1.5% agarose gel electrophoresis. The bands are located at approximately 450–480 bp (Figure 3C,D). The purified VH and VL gene fragments, each with restriction enzyme sites and a flexible linker, were pooled and used as templates for assembly by overlap extension PCR. The results indicate that the VH and VL fragments are approximately 480 bp and 450 bp long, respectively (Figure 4). Consequently, we linked them with a glycine–serine linker using overlap extension PCR. The scFvFgSAP2 library is approximately 930 bp (Figure 4). The scFvFgSAP2 library was ligated to the phagemid pCANTAB5E and used to transform the *E. coli* strain TG1 to select the scFvFgSAP2 with the strongest affinity for phage-binding to SAP2. The successful enrichment of target-specific phages across the panning rounds was validated by the progressive increase in phage titers, with detailed input and output numbers summarized in Table S1 (Supplementary Materials).

### 2.4. Sequence Analysis and Molecular Docking of scFvFgSAP2

**Figure 5:**
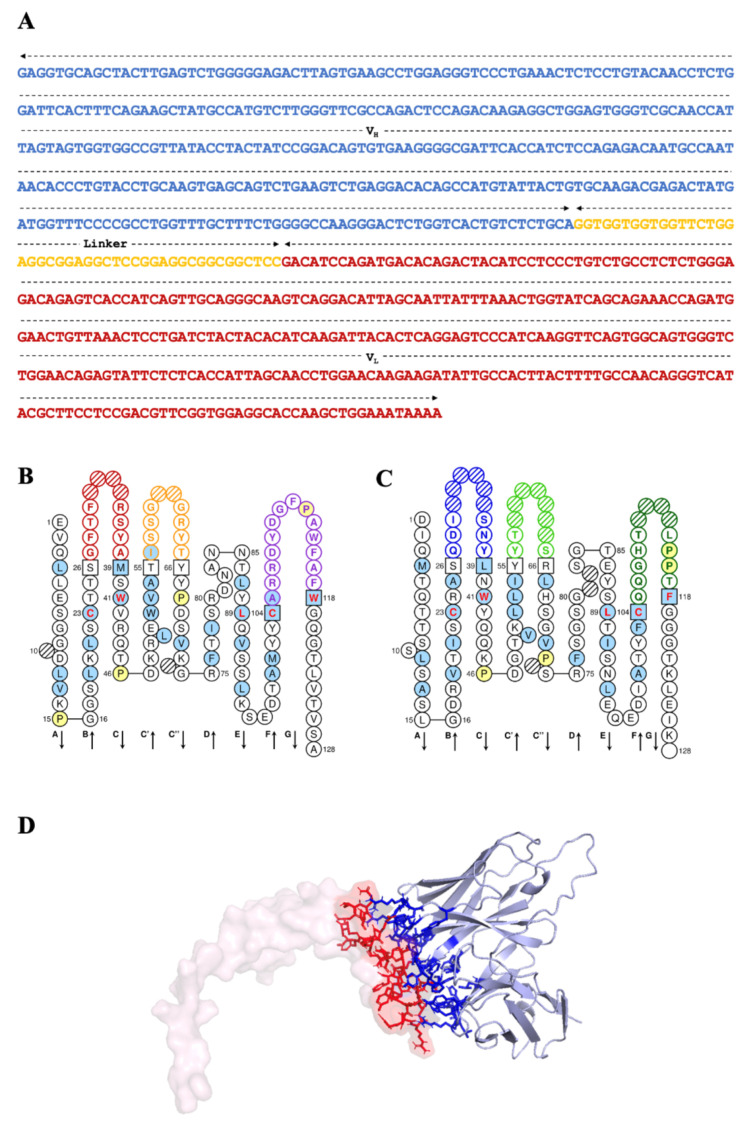
(**A**) Nucleotide sequence and immunoglobulin domain prediction of scFvFgSAP2. The scFvFgSAP2 consists of the variable heavy chain domain (VH, shown in blue) and the variable light chain domain (VL, shown in red), connected by a flexible glycine–serine linker (shown in yellow). (**B**,**C**) Two-dimensional graphical representations of the VH and VL protein domains, respectively. The 2D structural domain maps of VH and VL from scFvFgSAP2 were generated using IMGT/Collier-de-Perles. VH domain, CDR1 is marked in red, CDR2 in yellow, and CDR3 in purple; in the VL domain, CDR1 is marked in blue, CDR2 in light green, and CDR3 in dark green. All proline residues are displayed in yellow. IMGT anchors are shown in squares. Hatched circles indicate IMGT gaps according to the IMGT unique numbering for the V domain. Positions shown in red bold indicate the four conserved positions common to both V and C domains. Hydrophobic amino acids (hydropathy index with positive value: I, V, L, F, C, M, A) and tryptophan (W), when found at a given position in more than 50% of sequences, are highlighted with a blue background. Arrows indicate the direction of the β-strands and their designations in 3D structures. (**D**) Model and amino acid residues participating in the interaction of scFvFgSAP2 against FgSAP2. Computerized interactions between model FgSAP2 (translucent pink surface) and scFvFgSAP2 (gray, mainly beta-sheet). The protein interaction residues of FgSAP2 and scFvFgSAP2 display red and blue sticks, respectively.

The fragment with the strongest affinity for phage-binding to SAP2 was investigated by DNA sequencing (Figure 5A). The DNA sequencing analysis revealed a total sequence length of 729 base pairs (bp), encoding 243 amino acids. The scFvFgSAP2 sequence includes the VH domain (363 bp), the glycine–serine linker (45 bp), and the VL domain (321 bp). This sequence was submitted to GenBank under accession number PQ226778. The antigen-binding sites of scFvFgSAP2 were identified by the International Immunogenetics Information System (IMGT). Two-dimensional graphical representations of the VH and VL domains of scFvFgSAP2 were drawn using IMGT/Collier-de-Perles (Figure 5A,B). The complementarity-determining regions (CDRs 1, 2, and 3) of both the VH and VL domains were identified and numbered using the Kabat numbering scheme. Within the VH domain, CDR 1, CDR 2, and CDR 3 are located at amino acid positions 27–38, 56–65, and 105–117, respectively (Figure 5B). Similarly, in the VL domain, CDR 1, CDR 2, and CDR 3 are located at amino acid positions 27–38, 56–65, and 103–117, respectively (Figure 5C). In addition, the three-dimensional model of scFvFgSAP2 was constructed and docked targeting SAP2 using AlphaFold2 and HADDOCK 2.4, respectively. The interaction residue of the complex is shown in Figure 5D. The complex of scFvFgSAP2-SAP2 was selected as the best complex to predict binding affinity (KD) and binding free energy (∆G) using PRODIGY. The KD and ∆G were 4.6 × 10^−10^ M and −12.7 kcal·mol^−1^, respectively. The scFvFgSAP2 exhibited the lowest KD and ∆G values, indicating the strongest binding affinity to SAP2.

### 2.5. Recombinant scFvFgSAP2 Protein Expression

**Figure 6:**
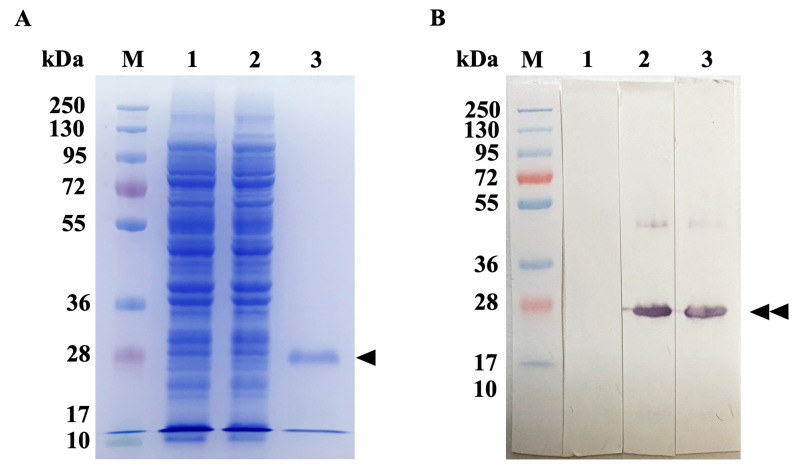
Purification and functional immunoblot analysis of the recombinant scFvFgSAP2. (**A**) SDS-PAGE analysis of the purified scFvFgSAP2 obtained via Ni-NTA affinity chromatography under native conditions, revealing a single prominent band at the expected molecular weight of approximately 27 kDa (Black triangle). (**B**) Immunoblot analysis using an anti-6xHis tag antibody confirms the identity of the purified approximately 27 kDa scFvFgSAP2 (Double black triangles).

The scFvFgSAP2 protein was expressed in a prokaryotic expression system induced with 1 M isopropyl β-D-thiogalactoside (IPTG) (final concentration at 1 mM) at 30 °C. The scFvFgSAP2 was purified under native conditions using Ni-NTA affinity chromatography. The purified scFvFgSAP2 was analyzed by SDS-PAGE and immunoblot analysis, which revealed that it had an approximate molecular weight of 27 kDa (Figure 6A,B).

### 2.6. Immunodetection of scFvFgSAP2 Against Parasite Native Proteins

**Figure 7:**
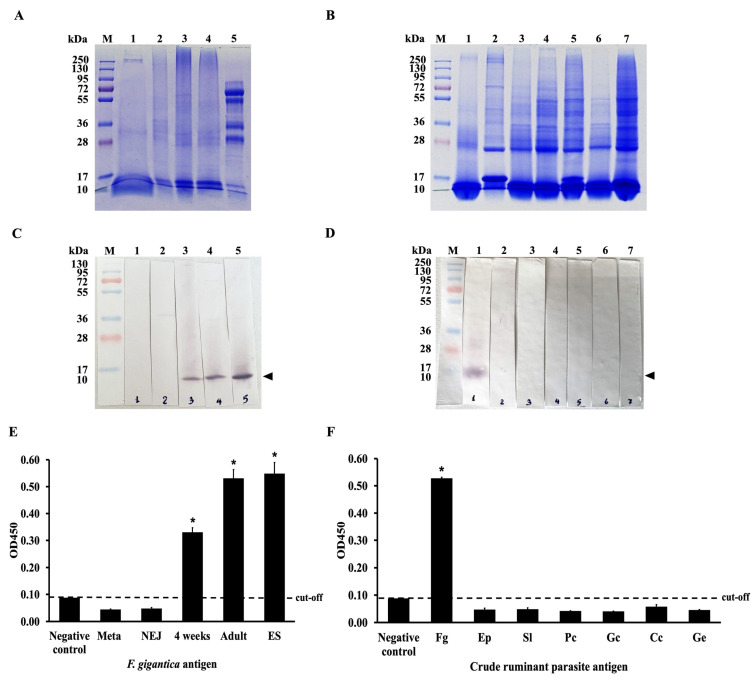
(**A**) Coomassie blue stained 12.5% SDS-PAGE gel of the WB of Metacercaria (Meta; lane 1), newsly excyst juveniles (NEJ; lane 2), 4-week-old juveniles (4WKJ; lane 3), adult stage (Adult; lane 4), and ES from adult *F. gigantica* (ES; lane 5). (**B**) Coomassie blue stained 12.5% SDS-PAGE gel of the WB of *F. gigantica* (Fg; lane 1) and other related parasites. *E. pancreaticum* (Ep; lane 2), *S. labiato-papillosa* (Sl; lane 3), *P. cervi* (Pc; lane 4), *G. crumenifer* (Gc; lane 5), *C. cotylophorum* (Cc; lane 6), and *G. explanatum* (Ge; lane 7). (**C**) Immunoblot analysis showing that scFvFgSAP2 reacted with *F. gigantica* at different developmental stages, including whole-body extracts of metacercaria (Meta; lane 1), newly excysted juveniles (NEJ; lane 2), 4-week-old juveniles (4 weeks; lane 3) and adults (lane 4), as well as adult ES (lane 5). The arrowhead indicates the FgSAP2 protein band. (**D**) Immunoblot analysis demonstrating scFvFgSAP2 strongly bound with whole-body extracts of *F. gigantica* (Fg; lane 1) and showed no cross-reactivity of scFvFgSAP2 with whole-body extracts of other parasites, including *E. pancreaticum* (Ep; lane 2), *S. labiato-papillosa* (Sl; lane 3), *P. cervi* (Pc; lane 4), *G. crumenifer* (Gc; lane 5), *C. cotylophorum* (Cc; lane 6), and *G. explanatum* (Ge; lane 7). The arrowhead indicates the FgSAP2 protein band. (**E**) Indirect ELISA showing that scFvFgSAP2 reacted with 4-week-old juveniles (4 weeks), adults, and adult ES of *F. gigantica* but not with metacercaria (Meta) or newly excysted juveniles (NEJ). (**F**) Indirect ELISA analysis confirmed no cross-reactivity with *E. pancreaticum* (Ep), *S. labiato-papillosa* (Sl), *P. cervi* (Pc), *G. crumenifer* (Gc), *C. cotylophorum* (Cc), and *G. explanatum* (Ge). Statistical analysis was performed using an independent *t*-test. Asterisks indicate statistically significant differences were observed when compared with the negative control (*p* value < 0.05).

The specificity and cross-reactivity of scFvFgSAP2 were investigated by immunoblotting. The scFvFgSAP2 bound specifically with whole-body extracts of 4-week-old juveniles, adults and adult ES products, respectively (Figure 7C). The specificity and cross-reactivity of scFvFgSAP2 were determined by indirect ELISA. It reacted strongly with whole-body extracts of 4-week-old juveniles and adults and ES products, respectively (Figure 7E). In addition, the cross-reactivity of scFvFgSAP2 with other ruminant parasite antigens—including *Eurytrema pancreaticum*, *Setaria labiato-papillosa*, *Paramphistomum cervi*, *Gastrothylax crumenifer*, *Cotylophoron cotylophorum*, and *Gigantocotyle explanatum*—was assessed using both immunoblotting and indirect ELISA. The results showed that scFvFgSAP2 strongly recognized a protein band of approximately 10 kDa in *F. gigantica*, while no cross-reactivity was observed with the other parasites (Figure 7D,F).

### 2.7. Immunolocalization Analysis

**Figure 8:**
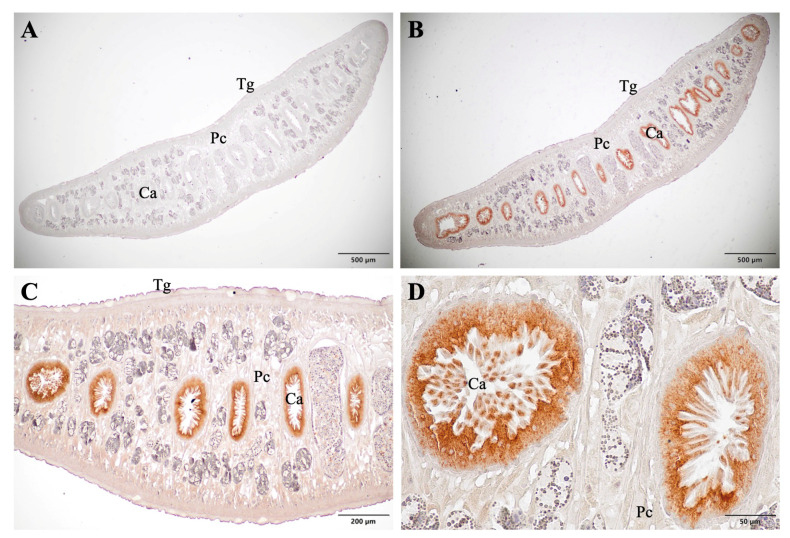
Immunolocalization of adult *F. gigantica* tissues using scFvFgSAP2 probes showed strong reddish staining and specificity in the cecum. (**A**) Negative control showing no staining; (**B**) low magnification (4×); (**C**) low magnification (10×); (**D**) high magnification (40×). In panels (**B**–**D**), intense staining was observed only in the cecal epithelial cells (Ca), with no staining detected in the tegument (Tg) or parenchyma (Pc).

Formalin-Fixed Paraffin-Embedded (FFPE) sections of adult *F. gigantica* were localized using scFvFgSAP2 to investigate its specificity for natural SAP2 in the parasite’s tissue. The immunolocalized sections were investigated and photographed using an OLYMPUS BX43 light microscope (Olympus, Shinjuku, Tokyo, Japan). The adult *F. gigantica* probed with scFvFgSAP2 exhibited strong reddish staining in the cecum, including the apical and cytoplasmic parts of the cecal epithelium cells (Figure 8B–D). In contrast, no staining was observed in the negative control sections, including the cecum, parenchyma, and tegument (Figure 8A). These findings indicate that scFvFgSAP2 specifically recognizes native SAP2 in the parasite tissues.

## 3. Discussion

The development of scFvFgSAP2 in this study represents a significant advancement in the field of *F. gigantica* research, particularly in comparison to previous studies. Prior attempts to develop specific diagnostic tools or therapeutic agents for fasciolosis have often been hampered by the cross-reactivity of antibodies with other parasites or the limited availability of specific antigens [39]. This study overcomes these challenges by utilizing a phage display library to select a highly specific scFv antibody with high affinity for SAP2, a unique antigen of *F. gigantica*. This approach offers several advantages, including the ability to rapidly screen large libraries of antibodies and select those with the desired specificity and affinity, surpassing traditional methods such as hybridoma technology [34,40,41,42,43,44,45]. Previous studies reported the development of monoclonal antibodies against various antigens of *F. gigantica*, including CatL [20], GSTs [39], and FABPs [26]. While these antibodies have shown promise as diagnostic tools, their specificity and sensitivity have often been limited due to cross-reactivity with other parasites or the low abundance of the target antigen in the parasite [46]. In contrast, the scFvFgSAP2 developed in this study demonstrates high specificity for *F. gigantica* across different life stages and does not cross-react with other ruminant parasites, as confirmed in an in vitro model using indirect ELISA and immunoblotting. We confirmed its binding affinity and specificity using an in silico model. AlphaFold2 was used to determine the three-dimensional structure of scFvFgSAP2, and HADDOCK 2.4 was used to dock the scFvFgSAP2 to the target, SAP2. PRODIGY was then used to evaluate the resultant scFvFgSAP2-SAP2 complex and predict the binding free energy (∆G) and binding affinity (KD). The predicted KD value was 4.6 × 10^−10^ M. The corresponding ∆G value was −12.7 kcal·mol^−1^. Therefore, in vitro and in silico models suggested that scFvFgSAP2 has high specificity and a strong binding affinity targeting the natural SAP2 in *Fasciola* spp. Furthermore, the strong binding affinity of scFvFgSAP2 to SAP2 suggests its potential as a diagnostic agent, a concept that has not been extensively explored in previous research on fasciolosis [7].

In recombinant antibody construction, several cloning strategies are available, ranging from traditional restriction enzyme-based cloning and TA cloning to more advanced, seamless techniques such as Gibson assembly or Gateway cloning. In this study, we utilized traditional restriction enzyme cloning and overlap extension PCR to construct the scFvFgSAP2 library. We chose this method because it provides a reliable, cost-effective, and highly efficient means of inserting the scFv sequence from the mouse immunoglobulin gene into the phage display library without introducing unwanted frameshifts. Our results evidenced successful construction of the recombinant phagemid vector with precise sequence fidelity. When compared to other studies developing recombinant antibodies for parasitic diagnostics, which are increasingly utilizing seamless cloning for high-throughput library construction [47], our traditional approach remains equally robust for the targeted expression of a selected single clone, ensuring high sequence fidelity and minimizing the amplification biases often encountered in complex library constructions [48]. Moreover, our method ensures high stability of the construct in the host cells, matching the construction efficiency reported in similar scFv development studies targeting parasite antigens [24]. Protein expression systems are broadly categorized as prokaryotic, such as *E. coli*, and eukaryotic expression systems (yeast, insect cells, and mammalian systems; HEK293 or CHO cells). In our study, we successfully produced the scFvFgSAP2 using a prokaryotic expression system (*E. coli*). The recombinant scFvFgSAP2 was subsequently purified using Ni-NTA affinity chromatography under native conditions. SDS-PAGE analysis revealed that the purified scFvFgSAP2 was highly pure, with a molecular weight of approximately 27 kDa. The primary advantages of the *E. coli* system are its rapid growth kinetics, cost-effectiveness, and ease of scaling, which are well-suited to the initial screening and production of recombinant antibodies. However, a notable limitation of this system is the lack of post-translational modifications; additionally, it has a very low yield of the soluble active form [49]. Furthermore, the small molecular size of the scFv makes it less suitable for certain downstream applications. To address these limitations, future work will focus on optimizing production by fusing scFvFgSAP2 with the IgG Fc region and expressing it in a eukaryotic expression system, such as HEK293 or CHO cells. This strategic shift will not only increase the production yield and molecular size but also facilitate proper folding and post-translational modifications, thereby enhancing its long-term stability and structural integrity [50,51,52,53].

The comprehensive characterization of scFvFgSAP2 via indirect ELISA, immunoblotting, and immunolocalization robustly confirmed its exceptional specificity, which can be categorized according to three key aspects: stage-specific, species-specific, and organ-specific targeting. Previous studies developing monoclonal antibodies against *F. gigantica* antigens (such as CatL, GST, and FABP) often report limited specificity and sensitivity due to cross-reactivity with other parasites or low target antigen abundance. In contrast, our in vitro indirect ELISA and immunoblot results demonstrated that scFvFgSAP2 has a highly species-specific profile that exhibits no cross-reactivity with other ruminant parasites. Furthermore, it revealed strong stage-specific recognition, effectively targeting *F. gigantica* across different critical life stages. Its specificity was further validated at the tissue level through immunolocalization studies, which demonstrated strict organ-specific binding to the cecum of adult *F. gigantica*. Because the cecum is the primary site of natural SAP2 expression [14], this localized binding confirms that scFvFgSAP2 can accurately target natural SAP2. While previous studies established a foundation for the development of novel diagnostics for fasciolosis, this study represents a significant step forward. The combination of high species, stage, and organ specificity, alongside the strong binding affinity of scFvFgSAP2 for SAP2, highlights its vast potential as a diagnostic agent—a concept that has not been explored extensively in existing fasciolosis research [17,18]. Future research should prioritize the optimization of scFvFgSAP2 production using eukaryotic systems and the translation of these findings into practical, field-ready diagnostic kits. Ultimately, this successful research could have a profound impact on the control, accurate surveillance, and prevention of fasciolosis in human and ruminant livestock.

## 4. Materials and Methods

**Figure 9:**
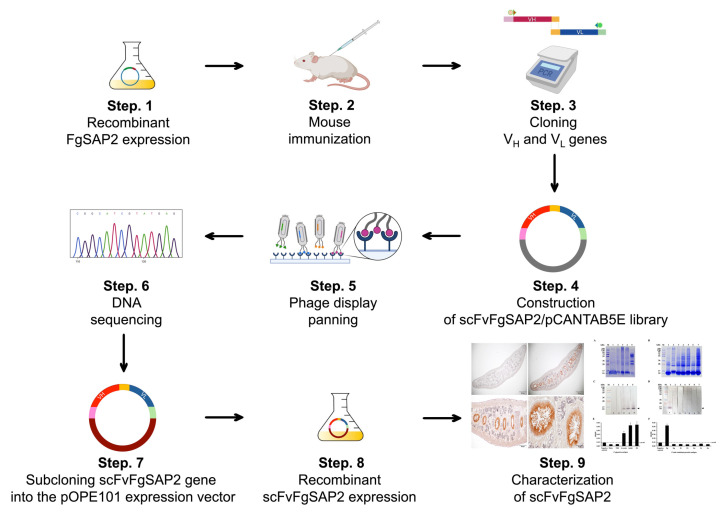
The overall workflow of this research consisted of nine sequential steps. First, recombinant rFgSAP2 was expressed (Step 1) and subsequently used for mouse immunization (Step 2). Following immunization, the variable heavy (VH) and light (VL) chain genes were amplified (Step 3) and cloned to construct an scFv library in the pCANTAB5E phagemid system (Step 4). Target-specific binders were then enriched and selected through phage display panning (Step 5), followed by identification of positive clones using DNA sequencing analysis (Step 6). The selected scFv genes were subsequently subcloned into the pOPE101 expression vector (Step 7) for soluble recombinant scFv expression (Step 8). Finally, the purified scFvFgSAP2 was subjected to downstream functional and binding characterization analyses (Step 9).

The overall workflow for this research investigation is shown in Figure 9.

### 4.1. Parasite Samples and Antigen Preparation

*F. gigantica* metacercariae were obtained from experimentally infected *Lymnaea ollula* snails. Newly excysted juveniles (NEJs) were generated by inducing metacercarial excystment, as previously described by Changklungmoa et al. (2018) [54]. Golden Syrian hamsters were experimentally infected with metacercariae, and 4-week-old juvenile parasites were recovered from their livers, as described previously [54]. Eggs and adult parasites were collected from cattle naturally infected with *F. gigantica* at a local abattoir in Pathum Thani Province, Thailand. The eggs were obtained from bile in the cattle’ gallbladders, while adult parasites were recovered from the bile ducts and gallbladders.

ES products of *F. gigantica* were prepared by incubating live adult parasites in RPMI 1640 and incubating at 37 °C for 3 h. Whole-body antigens (WBs) from *F. gigantica* (Fg), *E. pancreaticum* (Ep), *S. labiato-papillosa* (Sl), *P. cervi* (Pc), *G. crumenifer* (Gc), *C. cotylophorum* (Cc), and *G. explanatum* (Ge) were prepared by homogenizing adult parasites in PBS (pH 7.4). Homogenates were centrifuged at 10,000× *g* for 30 min at 4 °C, following the method of Changklungmoa et al. (2014) [55]. The resulting antigen-containing supernatants were collected and stored at −80 °C until use.

### 4.2. Recombinant FgSAP2 (rFgSAP2) Protein Production

The rFgSAP2 was obtained from the Research Unit for Vaccine and Diagnosis of Parasitic Diseases, Burapha University, Thailand, and prepared according to the protocol described previously [14]. Briefly, *E. coli* BL21(DE3) harboring the recombinant FgSAP2/pET-30b plasmid was cultured in Luria–Bertani (LB) medium containing 100 μg/mL. The rFgSAP2 expression was induced with isopropyl β-D-1-thiogalactopyranoside (IPTG; Merck, Darmstadt, Germany) at a final concentration of 1 mM. The rFgSAP2 was purified using Ni-NTA affinity chromatography and dialyzed against 0.1 M PBS (0.13 M NaCl, 0.002 M KCl, 0.01 M Na_2_HPO_4_, and 0.001 M KH_2_PO_4_; pH 7.0) [14]. Protein samples, including non-induced, induced, and purified rFgSAP2, were analyzed by SDS-PAGE. The purified rFgSAP2 was stored at −20 °C until use.

Confirmation of rFgSAP2 production by Western blot analysis. Protein samples, including non-induced, IPTG-induced, and purified rFgSAP2, were separated by 12.5% SDS-PAGE. Following electrophoresis, the proteins were electrotransferred onto a nitrocellulose membrane (Bio-Rad, Hercules, CA, USA) as previously described by Kueakhai et al. [14]. The membranes were washed three times with 0.1% PBS-T and blocked with 4% (*w*/*v*) non-fat skim milk for 30 min to prevent non-specific binding. After blocking, the membranes were incubated with rabbit anti-rFgSAP2 antibody [14] at room temperature for 2 h, followed by three washes with PBS-T. Subsequently, the membranes were incubated with alkaline phosphatase (AP)-conjugated goat anti-rabbit IgG (Invitrogen, Waltham, MA, USA), diluted 1:2000 in 0.1 M PBS, at room temperature for 1 h. The membranes were then washed thoroughly (five times) with 0.1% PBS-T. Signal detection was performed using nitro-blue tetrazolium chloride/5-bromo-4-chloro-3-indolyl phosphate (NBT/BCIP) substrate, and the reaction was terminated using stop buffer (0.01 M PBS containing 20 mM EDTA, pH 8.0).

### 4.3. Immunization Experiment in Mouse and Determination of Immune Response

A four-week-old BALB/c mouse was immunized subcutaneously with rFgSAP2 protein in 0.1 M PBS at a dose of 100 µg. For the primary immunization, 100 µg of rFgSAP2 protein was emulsified with complete Freund’s adjuvant (Sigma, St. Louis, MO, USA). The first and second booster immunizations were administered with 50 µg of rFgSAP2 protein emulsified with incomplete Freund’s adjuvant (Sigma, USA) [18]. Ten days after the second booster, blood was collected from the immunized mouse. Antibody levels in the serum were determined by indirect ELISA. The mouse was sacrificed, and its spleen was collected. This animal protocol was established in accordance with the Ethical Principles and Guidelines for the Use of Animals, as outlined by the National Research Council of Thailand, and was approved by the Animal Care and Use Committee of Burapha University (IACUC 020/2567).

The 96-well plates were coated with rFgSAP2 (1 µg/mL) in carbonate–bicarbonate buffer and incubated overnight at 4 °C. The plates were then washed three times with 0.05% PBS-T and blocked with 1% BSA in 0.1 M PBS for 1 h at room temperature with shaking to prevent non-specific binding. Subsequently, serially diluted serum samples in PBS (1:250–1:4,096,000) were added to the wells (50 µL/well), incubated with shaking for 2 h, and washed three times with 0.05% PBS-T. After washing, goat anti-mouse IgG (Invitrogen, USA) diluted 1:5000 in 0.1 M PBS was added to each well (50 µL/well) and incubated with shaking for 1 h, followed by three washes with 0.05% PBS-T. The plates were then incubated with horseradish peroxidase (HRP)-conjugated secondary antibody (Invitrogen, USA), diluted 1:5000 in 0.1 M PBS (50 µL/well), at room temperature for 1 h, and washed five times with 0.05% PBS-T. Color development was performed by adding 100 µL of TMB substrate (Sigma, USA) to each well and incubating in the dark for 10 min. The reaction was stopped by adding 1 N HCl, and the optical density (OD) was measured at 450 nm using a microplate reader (VERSAMAX, Molecular Devices, San Jose, CA, USA).

### 4.4. Cloning and Construction of scFv DNA Library

The mouse spleen was extracted for total RNA using the RNeasy Mini Kit (Qiagen, Germantown, MD, USA). The purified RNA was stored at −80 °C. First-strand cDNA was synthesized using a RevertAid First Strand cDNA Synthesis Kit (Thermo Fisher Scientific, Waltham, MA, USA). We cloned VH and VL domains using specific primer sets designed based on the mouse immunoglobulin gene database [56,57]. To capture the maximum immunological diversity of the murine antibody repertoire, an extensive set of multiplexed primer pairs was utilized for the amplification of VH and VL genes. Because the murine immune system utilizes numerous variable gene families, this comprehensive primer design is crucial to universally amplify the broad spectrum of VH and VL sequences. This strategic approach prevents the loss of potentially high-affinity clones and ensures the construction of a highly representative and diverse scFv library against SAP2. Briefly, the VH gene repertoires were amplified by PCR using a set of VH forward primers (MuVH1-MuVH15/2) paired with a mixture of JH reverse primers (MuJH1-MuJH4) (Table 1). The VL gene repertoires were amplified using a set of VL forward primers (VK1-VK13) paired with a mixture of JK reverse primers (JK1/2-JK5) (Table 1). All reaction tubes were performed with an initial denaturation at 98 °C for 1 min, followed by 35 cycles of 98 °C for 10 s, 60 °C for 10 s, and 72 °C for 20 s, and a final extension at 72 °C for 5 min. The reaction was then stopped and held at 4 °C. The purified VH and VL gene fragments were then used as templates for reamplification using restriction enzyme sites and a flexible linker. The purified VH gene fragments were reamplified using a set of forward primers containing an *Sfi*I site (MuVH1-*Sfi*I–MuVH15/2-*Sfi*I) and reverse primers incorporating a (G4S)2 linker (MuJH1-(G4S)2–MuJH4-(G4S)2). The VL gene fragments were reamplified using forward primers with a (G4S)2 linker (MuVK1-(G4S)2–MuVK13-(G4S)2) and reverse primers containing a *Not*I site (MuJK1/2-*Not*I–MuJK5-*Not*I). All reaction tubes were performed with an initial denaturation at 98 °C for 1 min, followed by 35 cycles of 98 °C for 10 s, 60 °C for 10 s, and 72 °C for 20 s, and a final extension at 72 °C for 5 min. The reaction was then stopped and held at 4 °C, and analyzed by 1.5% agarose gel electrophoresis. The gels were purified using a QIAquick^®^ Gel Extraction kit (Qiagen, Hilden, Germany). The purified VH and VL gene fragments, each with restriction enzyme sites and a flexible linker, were pooled and used as templates for assembly by overlap extension PCR. The PCR was performed with an initial denaturation at 98 °C for 1 min, followed by 25 cycles of 98 °C for 10 s and 72 °C for 30 s, and a final extension at 72 °C for 5 min. Subsequently, the scFvFgSAP2 gene was ligated into the pCANTAB5E phagemid vector (Creative Biolabs, Shirley, NY, USA; Figure S1) and transformed into the *E. coli* strain TG1 (GoldBio, St. Louis, MO, USA). Because the pCANTAB 5E phagemid vector lacks the full complement of structural genes required for viral assembly, the M13K07 helper phage was utilized to rescue the phagemid. The M13K07 helper phage infects the phagemid-harboring *E. coli* and provides the necessary phage packaging machinery and structural proteins. Due to a mutation in the helper phage’s origin of replication, the *E. coli* host preferentially packages the single-stranded phagemid DNA, which encodes the scFv-pIII fusion protein, into the assembled progeny phage particles.

### 4.5. Bio-Panning

A single clone of *E. coli* TG1 containing the pCANTAB5E/scFvFgSAP2 phagemid vector was chosen from a 2 XYT-ampicillin agar plate and cultured in 2XYT broth containing 100 μg/mL ampicillin at 37 °C with shaking 250 rpm until the optical density at 600 nm reached 0.5 to 0.7. Then, the bacterial culture was infected with 10^12^ pfu/mL of M13KO7 helper phages (NEB, Ipswich, MA, USA) at 37 °C with shaking for 2 h. After infection with the helper phages, fifty micrograms per milliliter of kanamycin was added to the culture and incubated at 37 °C with shaking at 250 rpm for 16 h. The culture was centrifuged, and the supernatant was transferred to a sterilized bottle. The supernatant was supplemented with PEG/NaCl at a ratio of one to five, and the phages were precipitated on ice for 1 h. The precipitated phages were then centrifuged at 10,000× *g* at 4 °C for 30 min, and the supernatant was discarded to remove as much PEG solution as possible. Finally, the pellet was suspended in 50% (*v*/*v*) glycerol in tris-buffered saline (TBS; 0.13 M NaCl, 0.002 M KCl, 0.099 M Tris, pH 8.6), and bio-panning was performed to select for the strongest binding affinity.

### 4.6. Sequence Analysis and Molecular Docking

The purified plasmid DNA, isolated from a single-isolated white colony, was purified (Quick Plasmid Miniprep Kit, Invitrogen, USA) for sequencing. DNA sequencing was verified by Macrogen, Seoul, Republic of Korea. Analysis of homologous sequences was performed using the international ImMunoGeneTics information system (IMGT) annotation and IMGT/Collier-de-Perles [58,59,60]. The amino acid sequences of scFvFgSAP2 and SAP2 were used to construct the three-dimensional structure using Alphafold2 (https://colab.research.google.com/github/sokrypton/ColabFold/blob/main/AlphaFold2.ipynb) (accessed on 1 April 2025) [61]. The antigen–antibody docking was investigated using HADDOCK 2.4 (https://wenmr.science.uu.nl/haddock2.4/) (accessed on 3 April 2025) [62]. Subsequently, the predicted binding affinity and free energy of the antigen–antibody complex were analyzed by PRODIGY (https://wenmr.science.uu.nl/prodigy/) (accessed on 3 April 2025) [63,64,65]. The antigen–antibody complex was visualized using the PyMOL program (https://www.pymol.org/) (accessed on 3 April 2025).

### 4.7. Expression and Purification of scFvFgSAP2 Protein

scFvFgSAP2 was produced using a prokaryotic expression system. Briefly, the *E. coli* strain HB2151, which contained the recombinant pOPE101 (PROGEN, Heidelberg, Germany; Figure S2)/scFvFgSAP2 plasmid, was inoculated into 5 mL of 2× YT medium containing 100 μg/mL ampicillin and incubated overnight at 37 °C with shaking at 250 rpm. The overnight culture was inoculated into 4 L of 2× YT medium containing 100 μg/mL ampicillin and incubated at 37 °C with shaking at 250 rpm; its optical density at 600 nm ranged from 0.5 to 0.7. The expression protein was induced by 1 mM IPTG at 30 °C with shaking at 250 rpm for 14–16 h and purified under native conditions by Ni-NTA affinity chromatography column (Qiagen, Heidelberg, Germany). Consequently, the scFvFgSAP2 was dialyzed with 0.1 M TBS, pH 8.0, containing 0.05% (*w*/*v*) NaN_3_ at 4 °C for 72 h and concentrated by centrifugal filter tube (Ultracel-3K, Amicon Ultra, Millipore, Darmstadt, Germany). The bacterial culture without 1 mM IPTG, the bacterial culture with 1 mM IPTG, and purified scFvFgSAP2 were analyzed by SDS-PAGE and immunoblot analysis.

### 4.8. Characterization of scFvFgSAP2 by ELISA Analysis

A ninety-six-well maxisorb microtiter plate was coated with crude *F. gigantica* antigen (1 µg/well), including metacercaria (Meta), newly excyst juveniles (NEJ), 4-week-old juveniles (4 weeks), adults (Adult), and ES with carbonate/bicarbonate buffer at 4 °C. To investigate specificity, we coated the plate using crude ruminant parasite antigens (1 µg/well) of *F. gigantica* (Fg), *E. pancreaticum* (Ep), *S. labiato-papillosa* (Sl), *P. cervi* (Pc), *G. crumenifer* (Gc), *C. cotylophorum* (Cc), and *G. explanatum* (Ge) with carbonate/bicarbonate buffer at 4 °C overnight. The plates were washed three times with 0.05% PBS-T and blocked for non-specific binding using 1% BSA in 0.1 M PBS with shaking at room temperature for 1 h. Subsequently, the scFvFgSAP2 in 0.1 M PBS was added to the well, incubated with shaking for 2 h, and washed three times with 0.05% PBS-T. After washing, rabbit anti-6xHis tag antibody (Invitrogen, USA) diluted in 0.1 M PBS (1:250) was added into the well, incubated with shaking for 1 h, and washed three times with 0.05% PBS-T. The plates were incubated with HRP-conjugated goat anti-rabbit IgG (H+L) antibody (Invitrogen, USA), diluted with 0.1 M PBS (1:5000) at room temperature for 1 h, and washed five times with 0.05% PBS-T. Color development was then performed by adding 100 μL of TMB substrate (Sigma, USA) to each well, followed by incubation in the dark for 10 min. The reaction was stopped by the addition of 1 N HCl, and the optical density was measured at 450 nm with a microplate reader machine (VERSAMAX, Molecular Devices, San Jose, CA, USA).

### 4.9. Characterization of scFvFgSAP2 by Immunoblot Analysis

Protein samples, including recombinant FgSAP2 (rFgSAP2; 5 µg), crude antigens from different developmental stages of *F. gigantica* (5 µg each)—Meta, NEJ, 4 weeks, adults, and ES products and crude ruminant parasite antigens (5 µg each), including *F. gigantica* (Fg), *E. pancreaticum* (Ep), *S. labiato-papillosa* (Sl), *P. cervi* (Pc), *G. crumenifer* (Gc), *C. cotylophorum* (Cc), and *G. explanatum* (Ge), were separated by 12.5% SDS-PAGE. Following electrophoresis, proteins were electrotransferred onto a nitrocellulose membrane (Bio-Rad, USA) using a wet/tank transfer system (Mini Trans-Blot, Bio-Rad, USA). The transfer was performed in a transfer buffer containing 25 mM Tris-HCl, 192 mM glycine, and 20% (*v*/*v*) methanol under the following conditions: 1 A for 45 min followed by 3 A for 15 min. After the transfer, the membranes were stained with Ponceau S solution (0.25% (*w*/*v*) Ponceau S in 1% (*v*/*v*) acetic acid) to confirm protein transfer and rinsed with deionized water to remove excess stain. The membranes were then washed three times with 0.1% PBS-T and blocked with 4% (*w*/*v*) non-fat skim milk for 30 min to prevent non-specific binding. The blocked membranes were incubated with recombinant scFvFgSAP2 diluted 1:500 in 0.1 M PBS at room temperature for 2 h, followed by three washes with PBS-T. Subsequently, the membranes were incubated with rabbit anti-6xHis tag antibody (Invitrogen, USA), diluted 1:250 in 0.1 M PBS, at room temperature for 1 h and washed three times. The membranes were then incubated with AP-conjugated goat anti-rabbit IgG (H+L) antibody (Invitrogen, USA), diluted 1:5000 in 0.1 M PBS, at room temperature for 1 h. After five washes with 0.1% PBS-T, signal detection was performed using NBT/BCIP substrate. The reaction was stopped with stop buffer (0.01 M PBS containing 20 mM EDTA, pH 8.0).

### 4.10. Characterization of scFvFgSAP2 by Immunolocalization Analysis

FFPE adult *F. gigantica* tissue was obtained from the Research Unit for Vaccine and Diagnosis of Parasitic Diseases, Burapha University, and sectioned at 5 µm thickness. The adult *F. gigantica* sections were de-paraffinized twice in xylene and rehydrated through a graded series of ethanol (absolute, 95%, 80%, and 70%). The sections were then rinsed in tap water and washed in 0.1 M PBS (pH 7.4) for 5 min. Antigen retrieval was performed by treating the sections three times with 10 mM citrate buffer (10 mM C_6_H_8_O_7_, pH 6.0). Endogenous peroxidase activity was blocked by incubating the sections in 3% (*v*/*v*) hydrogen peroxide prepared in 0.1 M PBS for 30 min at room temperature in the dark with gentle shaking, followed by rinsing in tap water for 5 min. Non-specific binding sites were blocked with 4% (*w*/*v*) bovine serum albumin (BSA) in 0.1 M PBS for 30 min. The sections were then incubated with scFv rFgSAP2 in PBS, while PBS alone (without scFv rFgSAP2) was used as a negative control, at room temperature for 2 h. After washing three times with PBS, the sections were incubated with rabbit anti-6×His tag antibody (Invitrogen, USA), diluted 1:200 in 0.1 M PBS, for 1 h at room temperature, followed by three washes. Subsequently, the sections were incubated with HRP-conjugated goat anti-rabbit IgG (H+L) antibody (Invitrogen, USA), diluted 1:2000 in 0.1 M PBS, for 1 h at room temperature. After five washes with 0.1% PBS-T, color development was performed using 3,3′-diaminobenzidine tetrahydrochloride (DAB) substrate solution (Merck, Darmstadt, Germany), and the sections were counterstained with Mayer’s hematoxylin. Finally, the sections were dehydrated through a graded ethanol series (70%, 80%, 95%, and absolute ethanol), cleared in xylene, and mounted with mounting medium. The prepared slides were examined and photographed using an OLYMPUS BX43 light microscope (Olympus, Shinjuku, Tokyo, Japan).

## 5. Conclusions

This study successfully developed scFvFgSAP2, a novel recombinant single-chain variable fragment targeting the unique SAP2 antigen of *F. gigantica*, utilizing robust phage display technology. Both in silico and in vitro characterizations demonstrated that scFvFgSAP2 has a strong binding affinity and high specificity for species, stage, and organ. While prokaryotic expression yielded a 27 kDa product with high purity, the strategic transition to a eukaryotic scFvFgSAP2-Fc expression system will resolve production and stability constraints. Ultimately, the remarkable specificity and structural viability of scFvFgSAP2 establish it as a robust, patentable core component for the development of an innovative, field-ready diagnostic kit, offering a highly accurate and scalable solution for the effective surveillance and control of fasciolosis in livestock.

## 6. Patents

The single-chain variable fragment against saposin-like protein 2 from liver fluke *Fasciola gigantica* in this study has been submitted for a patent at the Department of Intellectual Property, Ministry of Commerce, Thailand, with the submission number 2301005233.

## Figures and Tables

**Table 1:** Degenerated primers for amplification and assembly of scFvFgSAP2.

Primer Name	Degenerative Primer Sequence (5′ → 3′)
**cDNA synthesis**	
MuIgG1/2-Rev	CTGGACAGGGATCCAGAGTTCC
MuIgG3-Rev	CCGCTGGACAGGGCTCCATAGTTCCA
MuCk-Rev	CTCATTCCTGTTGAAGCTCTTGAC
**V_H_ amplification**	
MuVH1-For	GAGGTTCDSCTGCAACAGTY
MuVH2-For	CAGGTGCAAMTGMAGSAGTC
MuVH3-For	GAVGTGMWGCTGGTGGAGTC
MuVH4-For	CAGGTTAYTCTGAAAGAGTC
MuVH5-For	GAKGTGCAGCTTCAGSAGTC
MuVH6-For	CAGATCCAGTTSGYGCAGTC
MuVH7-For	CAGRTCCAACTGCAGCAGYC
MuVH8-For	GAGGTGMAGCTASTTGAGWC
MuVH9-For	GAAGTGAAGMTTGAGGAGTC
MuVH10-For	GATGTGAACCTGGAAGTGTC
MuVH11-For	CAGATKCAGCTTMAGGAGTC
MuVH12-For	CAGGCTTATCTGCAGCAGTC
MuVH13-For	CAGGTTCACCTACAACAGTC
MuVH14-For	CAGGTGCAGCTTGTAGAGAC
MuVH15-For	GARGTGMAGCTGKTGGAGAC
MuVH15/2-For	GAGGTGCAGCTGGTGGAATC
MuJH1-Rev	GAGGAGACGGTGACCGTGGTCCC
MuJH2-Rev	GAGGAGACTGTGAGASTGGTGCC
MuJH3-Rev	GCAGAGACAGTGACCAGAGTCCC
MuJH4-Rev	GAGGAGACGGTGACTGAGGTTCC
**V_L_ amplification**	
MuVK1-For	CGACAWTGTTCTCACCCAGTC
MuVK2-For	CGACATCCAGATGACACAGWC
MuVK3-For	CGATRTTGTGATGACCCAGWC
MuVK4-For	CGACATTSTGMTGACCCAGTC
MuVK5-For	CGATGTTKTGVTGACCCAAAC
MuVK6-For	CGACACAACTGTGACCCAGTC
MuVK7-For	CGAYATTKTGCTCACTCAGTC
MuVK8-For	CGATATTGTGATRACCCAGGM
MuVK9-For	CGACATTGTAATGACCCAATC
MuVK10-For	CGACATTGTGATGWCACAGTC
MuVK11-For	CGATRTCCAGATGAMCCAGTC
MuVK12-For	CGATGGAGAAACAACACAGGC
MuVK13-For	CSAAAWTGTKCTCACCCAGTC
MuJK1/2-Rev	GCGTTTBATTTCCAGCTTGG
MuJK3-Rev	GCGTTTTATTTCCAGTCTGG
MuJK4-Rev	GCGTTTTATTTCCAAYTTTG
MuJK5-Rev	GCGTTTCAGCTCCAGCTTGG
**V_H_ reamplification**	
MuVH1-*Sfi*IFor	**ACTGCGGCCCAGCCGGCCATGGCC**GAGGTTCDSCTGCAACAGTY
MuVH2-*Sfi*IFor	**ACTGCGGCCCAGCCGGCCATGGCC**CAGGTGCAAMTGMAGSAGTC
MuVH3-*Sfi*IFor	**ACTGCGGCCCAGCCGGCCATGGCC**GAVGTGMWGCTGGTGGAGTC
MuVH4-*Sfi*IFor	**ACTGCGGCCCAGCCGGCCATGGCC**CAGGTTAYTCTGAAAGAGTC
MuVH5-*Sfi*IFor	**ACTGCGGCCCAGCCGGCCATGGCC**GAKGTGCAGCTTCAGSAGTC
MuVH6-*Sfi*IFor	**ACTGCGGCCCAGCCGGCCATGGCC**CAGATCCAGTTSGYGCAGTC
MuVH7-*Sfi*IFor	**ACTGCGGCCCAGCCGGCCATGGCC**CAGRTCCAACTGCAGCAGYC
MuVH8-*Sfi*IFor	**ACTGCGGCCCAGCCGGCCATGGCC**GAGGTGMAGCTASTTGAGWC
MuVH9-*Sfi*IFor	**ACTGCGGCCCAGCCGGCCATGGCC**GAAGTGAAGMTTGAGGAGTC
MuVH10-*Sfi*IFor	**ACTGCGGCCCAGCCGGCCATGGCC**GATGTGAACCTGGAAGTGTC
MuVH11-*Sfi*IFor	**ACTGCGGCCCAGCCGGCCATGGCC**CAGATKCAGCTTMAGGAGTC
MuVH12-*Sfi*IFor	**ACTGCGGCCCAGCCGGCCATGGCC**CAGGCTTATCTGCAGCAGTC
MuVH13-*Sfi*IFor	**ACTGCGGCCCAGCCGGCCATGGCC**CAGGTTCACCTACAACAGTC
MuVH14-*Sfi*IFor	**ACTGCGGCCCAGCCGGCCATGGCC**CAGGTGCAGCTTGTAGAGAC
MuVH15-*Sfi*IFor	**ACTGCGGCCCAGCCGGCCATGGCC**GARGTGMAGCTGKTGGAGAC
MuVH15/2-*Sfi*IFor	**ACTGCGGCCCAGCCGGCCATGGCC**GAGGTGCAGCTGGTGGAATC
MuJH1-(G_4_S)_2_Rev	GGAGCCTCCGCCTCCAGAACCACCACCACCTGAGGAGACGGTGACCGTGGTCCC
MuJH2-(G_4_S)_2_Rev	GGAGCCTCCGCCTCCAGAACCACCACCACCTGAGGAGACTGTGAGASTGGTGCC
MuJH3-(G_4_S)_2_Rev	GGAGCCTCCGCCTCCAGAACCACCACCACCTGCAGAGACAGTGACCAGAGTCCC
MuJH4-(G_4_S)_2_Rev	GGAGCCTCCGCCTCCAGAACCACCACCACCTGAGGAGACGGTGACTGAGGTTCC
**V_L_ reamplification**	
MuVK1-(G_4_S)_2_For	GGAGGCGGAGGCTCCGGAGGCGGCGGCTCCGACAWTGTTCTCACCCAGTC
MuVK2-(G_4_S)_2_For	GGAGGCGGAGGCTCCGGAGGCGGCGGCTCCGACATCCAGATGACACAGWC
MuVK3-(G_4_S)_2_For	GGAGGCGGAGGCTCCGGAGGCGGCGGCTCCGATRTTGTGATGACCCAGWC
MuVK4-(G_4_S)_2_For	GGAGGCGGAGGCTCCGGAGGCGGCGGCTCCGACATTSTGMTGACCCAGTC
MuVK5-(G_4_S)_2_For	GGAGGCGGAGGCTCCGGAGGCGGCGGCTCCGATGTTKTGVTGACCCAAAC
MuVK6-(G_4_S)_2_For	GGAGGCGGAGGCTCCGGAGGCGGCGGCTCCGACACAACTGTGACCCAGTC
MuVK7-(G_4_S)_2_For	GGAGGCGGAGGCTCCGGAGGCGGCGGCTCCGAYATTKTGCTCACTCAGTC
MuVK8-(G_4_S)_2_For	GGAGGCGGAGGCTCCGGAGGCGGCGGCTCCGATATTGTGATRACCCAGGM
MuVK9-(G_4_S)_2_For	GGAGGCGGAGGCTCCGGAGGCGGCGGCTCCGACATTGTAATGACCCAATC
MuVK10-(G_4_S)_2_For	GGAGGCGGAGGCTCCGGAGGCGGCGGCTCCGACATTGTGATGWCACAGTC
MuVK11-(G_4_S)_2_For	GGAGGCGGAGGCTCCGGAGGCGGCGGCTCCGATRTCCAGATGAMCCAGTC
MuVK12-(G_4_S)_2_For	GGAGGCGGAGGCTCCGGAGGCGGCGGCTCCGATGGAGAAACAACACAGGC
MuVK13-(G_4_S)_2_For	GGAGGCGGAGGCTCCGGAGGCGGCGGCTCCSAAAWTGTKCTCACCCAGTC
MuJK1/2-*NotI*Rev	**GAGTCATTCTCGACTTGCGGCCGC**GCGTTTBATTTCCAGCTTGG
MuJK3-*Not*IRev	**GAGTCATTCTCGACTTGCGGCCGC**GCGTTTTATTTCCAGTCTGG
MuJK4-*Not*IRev	**GAGTCATTCTCGACTTGCGGCCGC**GCGTTTTATTTCCAAYTTTG
MuJK5-*Not*IRev	**GAGTCATTCTCGACTTGCGGCCGC**GCGTTTCAGCTCCAGCTTGG

The underlined sequence represents the linker, while the bold sequence indicates the restriction site.

## Data Availability

The raw data for DNA sequencing are deposited in the NCBI Nucleotide database under GenBank accession number PQ226778.

## Supplementary Materials

The following supporting information can be downloaded at: https://www.mdpi.com/article/10.3390/ijms27104474/s1.

## Abbreviations

The following abbreviations are used in this manuscript:

∆G	Binding free energy
DAB	3, 3′ diaminobenzidine tetrahydrochloride
ELISA	Enzyme-linked immunosorbent assay
ES	Excretory-secretory
FFPE	Formalin-fixed paraffin-embedded
KD	Binding affinity
IgG	Immunoglobulin G
IMGT	International immunogenetics information system
IPTG	Isopropyl β-D-thiogalactoside
PBS	Phosphate-buffered saline
PBS-T	Phosphate-buffered saline-Tween20
rFgSAP2	Recombinant saposin-like protein 2 from the liver fluke F. gigantica
SAPs	Saposin-like proteins
SAP2	Saposin-like protein 2
scFv	Single-chain variable fragment
SDS-PAGE	Sodium Dodecyl Sulfate–Polyacrylamide Gel Electrophoresis
TBS	Tris-buffered saline
VH	Variable heavy chain
VL	Variable light chain
