# Supplementary material for: Production and Characterization of Recombinant Single-Chain Variable Fragment (scFv) Antibody Against Fasciola gigantica Saposin-like Protein 2
Source: Int J Mol Sci. 2026 May 16;27(10):4474. doi: 10.3390/ijms27104474 (PMC13208075; doi:10.3390/ijms27104474)
Supplement: Supplementary file 1 [file ijms-27-04474-s001.zip › ijms-4229548-supplementary.pdf]

Figure S1. Schematic diagram of the pCANTAB5E phagemid vector (Amersham Biosciences, UK). The vector is designed for the cloning, expression, and phage display of recombinant antibodies. The top panel illustrates the insertion strategy for a single-chain variable fragment (scFv), comprising heavy (VH) and light (VL) chain domains joined by a flexible linker, using the SfiI and NotI restriction sites. Transcription is driven by the inducible lac promoter (Plac), followed by a g3 signal sequence that directs the expressed protein to the bacterial periplasm. Downstream of the NotI site, the construct incorporates an E-tag for immunodetection and an amber stop codon preceding the fd phage gene III. This amber codon enables expression of the scFv as a fusion protein with the minor coat protein (pIII) in suppressor *E. coli* strains (TG1) for phage display, or as a soluble non-fused scFv in non-suppressor strains (HB2151). The phagemid backbone also contains an ampicillin resistance gene (Amp^{r}) for bacterial selection, a ColE1 origin of replication for propagation in *E. coli*, and an M13 origin for packaging into single-stranded phage particles following helper phage rescue.

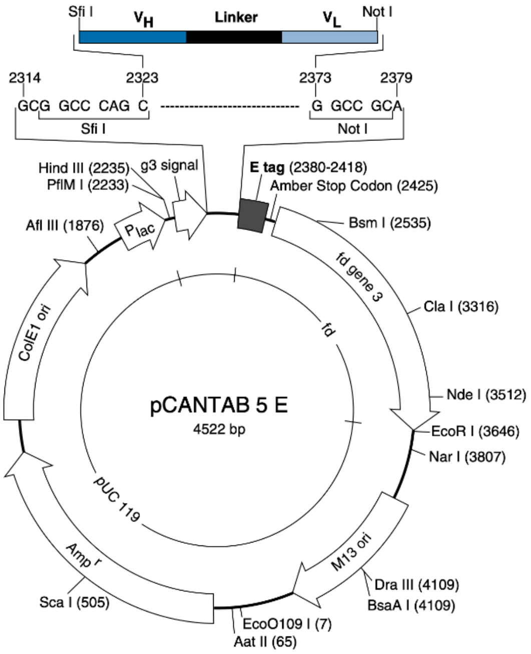

Figure S2. Schematic diagram of the pOPE101 expression vector (PROGEN, Germany). The upper panel presents the circular vector backbone, which contains a synthetic promoter (P/A1/04/03) for transcriptional regulation, a T7 terminator, an ampicillin resistance gene (Amp) for bacterial selection, and an origin of replication (ori). The dotted arc indicates the insertion region located between the EcoRI and XbaI restriction sites. The lower panel illustrates the linear arrangement of the cloned expression cassette encoding a single-chain variable fragment (scFv). From the 5′ to 3′ end, the cassette comprises a ribosome binding site (RBS), a PelB leader sequence for periplasmic secretion, a Serum A epitope, the heavy chain variable domain (VH), a Yol1/34 epitope functioning as a linker, the light chain variable domain (VL), a c-Myc epitope (MAb Myc 1-9E10), and a hexahistidine tag (6×His) for downstream detection and purification, followed by a stop codon. Relevant restriction endonuclease sites used for cloning and cassette modularity are indicated above the sequence.

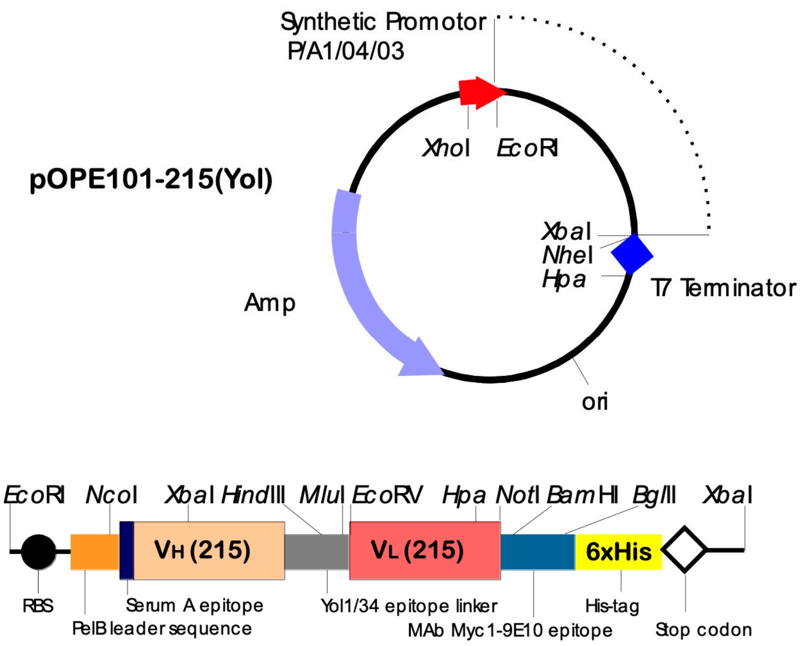

Table S1. Phage titer result of each panning round.

Round of panning	rFgSAP2 coating (µg/mL)	Number of washing	Input phage	Output phage
1	10	10	1 x 10^{11}	6.44 x 10^{6}
2	1	20	1 x 10^{11}	5.04 x 10^{8}
3	1	30	1 x 10^{11}	2.81 x 10^{8}
